# Perceived barriers and facilitators for adhering to COVID-19 preventive measures in Chile: a qualitative study in three large cities

**DOI:** 10.1186/s12879-023-08118-x

**Published:** 2023-03-14

**Authors:** Alexandra Obach, Báltica Cabieses, Francisca Vezzani, Consuelo Robledo, Alice Blukacz, Pablo Vial

**Affiliations:** 1 Instituto de Ciencias e Innovación en Medicina (ICIM), Facultad de Medicina Clínica Alemana, Universidad del Desarrollo, Santiago, Chile; 2 Department of Health Sciences, University of York, York, UK

**Keywords:** COVID-19, Prevention, Adherence to treatments, Social Determinants of Health, Chile, Latin America, Qualitative

## Abstract

**Background:**

Globally, it has been reported that different social determinants of health -structural, sociodemographic, economic, living conditions and cultural factors- may affect opportunities to adhere to prevention measures against SARS-CoV-2. The objective of this study was to explore the perceptions around barriers and facilitators for adherence to COVID-19 prevention measures among the adult population residing in three large cities in Chile from a social determinants of health perspective.

**Methods:**

Qualitative paradigm, multiple case-study design. Online semi-structured interviews were conducted with men and women aged 18 and over from different socioeconomic groups residing in three large cities. For participant recruitment and selection, purposive contacts were made based on community and social media networks, followed by snowball sampling. Saturation was reached at 61 participants, after which a thematic analysis was carried out with the support of AtlasTi software. The Ethics Committee of the Universidad del Desarrollo in Chile approved this study.

**Results:**

The main perceived barriers to adherence to COVID-19 preventive measures are linked to structural social determinants of health such as income, occupation, gender, access to basic supplies, and housing. Perceived facilitators are the fear of contagion and the incorporation of measures into daily habits. The social communication of preventive measures by health authorities is perceived as punitive, affecting adherence once the fear of contagion decreased in the country. It is also perceived that the recommended preventive measures are disconnected from communities’ cultural practices and people´s identity, as well as affected by gender inequities and socioeconomic conditions that stakeholders in the country do not sufficiently address.

**Conclusion:**

Study findings suggest that adherence to preventive measures, such as social distancing, mask use, and hand washing, could be promoted through their incorporation into the daily life habits of people and communities. These measures should consider the structural social determinants that generate multiple barriers to adherence, like poverty, occupational risks, and overcrowding. Socio-cultural dimensions of health and everyday risks need further understanding among the different communities in the country, allowing for differences in viewpoints and practices based on gender, age, place, and social identity.

## Background

The health crisis generated by the SARS-CoV-2 pandemic has implicated significant challenges for societies, States, and health systems. Efforts to curb the pandemic have focused mainly on implementing a range of non-pharmacological prevention measures, such as hand washing, physical distancing, use of masks, quarantines, contact tracing and border control. However, to make the compliance of these measures sustainable over time and to assess their relevance for future health crises, several authors emphasise the need to identify barriers and facilitators for adherence to preventive and control measures. This is key to ensuring that public health interventions implemented in future health crises such as global pandemics come from robust evidence demonstrating their effectiveness in real-life settings [1].

International evidence indicates that to analyze the compliance of COVID-19 preventive health measures, it is necessary to consider the Social Determinants of Health (SDH), defined as the situations and contexts of life and work that can influence health [2]. At the global level, it has been reported that various SDH, including structural factors -public policies and regulations-, socioeconomic factors (e.g., income, occupation) and living conditions, as well as cultural factors, affect the ability of populations to adhere to the sanitary measures established by health authorities [3]. For example, the existing evidence at the international level indicates that being a woman, over 18 years old and residing in urban areas are factors associated with adequate adherence to preventive and control measures [1, 4–17]. On the other hand, being young, male, and living in rural areas are factors associated with lower knowledge levels and lower adherence to prevention measures [7, 8, 15–18]. Likewise, evidence suggests that having a higher income, being employed, and having health insurance entail better adherence to preventive behaviors [13, 19–22].

Regarding educational factors, positive associations are reported between a higher level of education [5, 11, 21–24], a higher degree of knowledge of the disease and adherence to preventive measures [10, 25, 26]. On the contrary, those without updated information about the condition or are skeptical about the disease or its contagion mechanisms are less likely to adhere [13, 14]. People with a higher self-perception of risk concerning getting the disease have also been found to be more adherent to preventive practices [8, 24, 26]. Concerning living conditions, people living in overcrowded households and homeless are less adherent to preventive COVID-19 practices. Evidence also reports that ethnic minorities, migrants, and refugees could be at greater risk of living in these precarious household conditions [27, 28]. Finally, it has been suggested that living with older adults is associated with stricter preventive measures [12], while being a university student or staying in student residences was identified as a risk factor for getting the disease [29].

Most studies about the transmission and contagion prevention measures of SARS-CoV-2 have been focused in North America, Europe and Asia. The implications of the pandemic in South America have been less attended [30]. In 2021, this region had 32.1% of the total deaths from COVID-19 reported worldwide, while its population represents only 8.4% of the world population. Chile, with a population of 17.574.003 persons [31], presents one of the worst indicators of the region in the association between overcrowding and excess of deaths in 2020 (R² = 0.158), followed by Brazil (R² = 0.150) and Costa Rica (R² = 0.073) [32]. As a country context that allows understanding of these health outcomes, it should be noted that in the last decades, Chile have experienced unprecedented development since the return to democracy, achieving economic, political and social stability. Focusing on the lower-income population, social policies have managed to reduce poverty in the country in all its regions. However, Chile is currently the second most unequal country in the OECD, with the income of the richest 20% of the population being ten times higher than that of the poorest quintile [33]. According to data from 2020, 10,8% of the population of the country lives in conditions of poverty or extreme poverty [34], 27.3% of the population has an informal occupation [31], and 6.5% of the homes have some degree of overcrowding [34].

Shortly after the World Health Organization (WHO) declared the COVID-19 epidemic a global pandemic in March 2020, the Chilean government decreed a State of Constitutional Exception for Catastrophe, which lasted for approximately one year. The aim was to have greater control over people’s transit and enforce compliance with quarantine and other social distancing measures. Among the different measures adopted, all of them aimed at curbing the transmission of SARS-CoV-2, some addressed the provision of essential services to low-income families, others promoted labor benefits to enable working from home, social benefits, closure of educational establishments and their later reopening, to mention the most significant ones. Regarding the containment measures for COVID-19, the “Step by Step Plan, we take care of ourselves” was designed and implemented, establishing mobility restrictions according to the phases of the plan [30]. These phases were the following: (1) total quarantine, (2) partial quarantine, and (3) partial opening, while the fourth and fifth phases meant a return to most “normal” activities [31]. However, regardless of the Step by Step Plan phase, health authorities maintained mass media communication of preventive measures during all waves of the pandemic in the country, and full compliance was expected (e.g., hand washing, physical distancing, use of face masks).

One significant measure adopted within the healthcare system was the establishment of the Chilean Health Network that coordinated public, private, and institutional establishments to increase the number of hospital beds to treat COVID-19 patients [35]. In this way, the Chilean health system, which is fragmented between the public and the private sector, meaning access to quality healthcare is conditional on income, temporarily overcame this structural shortcoming to generate a nationwide network of respiratory ventilators in ICU beds across both systems. As a result, by February 2021, the country had experienced four waves of infections, with 1,590,887 accumulated infections as of July 13 [36]. Likewise, that same year in February, the population vaccination process began with a progressive reduction in respiratory ventilator requirements nationwide.

Despite the efforts made by the government, the measures did not impact the population in the same way. In Santiago, it has been reported that poorer boroughs have both higher incidence and higher mortality rates related to COVID-19 compared to more affluent boroughs [30]. Likewise, mobility during quarantine was reduced by 61% in the most affluent boroughs, while it was only reduced by 40% in the poorest boroughs. It has also been reported that boroughs with a higher socioeconomic level documented a more significant number of tests (PCR) carried out than in less affluent boroughs. In the latter, there were more significant delays in the delivery of results [30].

From the perspective of risk communication, it has been suggested that it is important not only to deliver the necessary and timely information so that citizens can make evidence-based decisions related to COVID-19 prevention, but also to understand their perceptions, beliefs and concerns about the pandemic and to incorporate this information into ongoing prevention strategies [37]. In this sense, the SDH approach, which coherently and systematically integrates different determinants related to possible barriers and facilitators for adherence to COVID-19 preventive measures, can be helpful in an investigative approach to this topic. However, evidence on barriers and facilitators for adhering to COVID-19 preventive measures based on the SDH perspective -accounting for individual and structural determinants of health, including socioeconomic and cultural dimensions- remains to be limited and further investigation is needed. In this context, the objective of this study was to explore the perceptions around barriers and facilitators for the adherence to the COVID-19 prevention measures promoted by the health authority in Chile among the adult population residing in three large urban cities: Santiago, Valparaíso, and Concepción, from the SDH perspective.

## Methods

### Study design

Qualitative, descriptive, and exploratory study [38], carried out between March and August 2021, which aimed at developing an in-depth understanding of the meanings that people give to the health measures promoted by the authorities for the containment of COVID-19 in the country, identifying the barriers and facilitators for adherence to these with the SDH perspective. A methodological design of multiple case-study [39] was carried out to address multiple cases, namely the barriers and facilitators for adhering to COVID-19 preventive measures by adults in the territories of Santiago, Valparaíso, and Concepción. These three cities were selected for being the most inhabited urban areas of the national territory [31].

### Participants recruitment and selection

The sample was constituted based on theoretical and feasibility criteria, considering areas of residence and socioeconomic group (SEG), based on household income. Theoretical sampling refers to the a priori establishment of categories of participant profiles according to the relevant dimensions of the SDH approach, which for this particular study were the following: age group, sex, socioeconomic level, and type of health insurance. The inclusion criteria for the sample were being over 18 years of age, residing in any of the three cities included the study, and speaking Spanish fluently (relevant for foreigner participants). The exclusion criteria were two: (i) that the potential participant had some physical or mental disability (intellectual, auditory or speech limitation) that limited his/her ability to decide to participate or to answer the interview, and (ii) no access to the Internet to conduct the interview. Study participants were recruited according to their borough of residency from the three large cities included in the study. Several boroughs were selected in each city to obtain a wide representation of SEG in each urban area. Selecting several boroughs in each city is due to the fact that in Chile there is a strong socioeconomic segregation between boroughs of residence. For the same reason, and to approach the experiences of adherence to Covid-19 prevention measures of different socioeconomic profiles, we emphasized the inclusion of different boroughs in each city addressed by the study. The SEG’s included in this study were defined from a descriptive borough-level analysis we conducted from the national representative CASEN survey in Chile (2017), ranking them according to their average income poverty index. Through this process, 6–8 boroughs from each of the cities of Santiago, Valparaíso and Concepción were selected.

For participant recruitment and selection, purposive contacts within each borough were made (i.e., seed contacts) based on community and social media networks established by the research team in previous projects, after which snowball sampling followed. Also, through connections with social organizations in each city, especially those linked to survival in the pandemic period, like community pots to feed the most vulnerable boroughs and municipal offices. People contacted through these strategies (and who met the inclusion criteria) were asked to participate by email or whatsapp. Those who expressed interest were then invited to receive a phone call from the project coordinators to learn more about the project and schedule an interview date. This process took place between March and April 2021. The initial sample size considered following the pre-established theoretical criteria was 60 people in all three selected cities (minimum of 20 per city, allowing for variations in sex, age, SEG and type of health insurance). This initial sample size was established considering that qualitative research seeks depth of information rather than a statistical representation of its sample [39], so it was considered that the minimum initial sample size of 20 participants should secure the expected depth of information necessary for this study. After data collection was finalized, the need for additional participants was explored through descriptive analysis, in which information saturation was found in all relevant study dimensions. As saturation of information for all main dimensions of interest was reached (COVID-19 preventive measures), we kept the sample size and only added one more for the city of Santiago, based on an additional participant who was willing to participate in the interview. Altogether, the final sample size was 61: 21 from Santiago, 20 from Valparaíso and 20 from Concepción. Participants are characterized around the following categories: (i) young men and women (18–24 years old), (ii) adult men and women (25–65 years old), and (iii) older men and women; all of them from different socioeconomic levels (based on the GFK/Adimark classification by household income: ABC1a-C1b, C2-C3, D-E, where group ABC1 corresponds to the highest segment, while group E to the lowest. This SEG nomenclature is defined based on the equivalent per capita income bracket, education level of the head or main household provider, and occupational level of the head or main household provider) [40, 41], with different types of health insurance (public and private), and from the three large urban areas selected for this study.

### Data collection techniques and instruments

Semi-structured interviews were conducted [39]. The information was collected between March and June 2021 by trained interviewers. Given the global emergency context resulting from the COVID-19 pandemic, the interviews were conducted online mainly through the virtual platform Zoom. The interviews lasted an average of 45 min (range of 35–60 min) and were based on a pre-defined interview script, which considered the following general categories: overall perceptions of experiences in relation to COVID-19; beliefs and perceptions around COVID-19 and the preventive measures recommended by health authorities; barriers and facilitators to adhering to measures to curb COVID-19 that were recommended by the health authority in the country at the time the study was conducted (COVID-19 preventive measures: social distancing, quarantining, use of masks, and hand washing; COVID-19 control measures: testing and traceability). The first ten interviews were developed by two interviewers to check the quality and reinforce standardized facilitation skills to ensure study objectives would be reached through the interviews. An exclusive professional Zoom account was used to protect the confidentiality of the conversation. For people without access to the platform, the interviews were carried out by Meet, Teams, or WhatsApp video call (used in less than ten interviews), recording the conversation through a digital audio recorder. All information was securely kept on the personal computer of the principal investigator.

### Data analysis and processing method

The information gathered through the semi-structured interviews was transcribed verbatim into Word. Each interview was assigned a code to protect participant confidentiality. The interview codes were created based on the following general structure: the interview number, the initial letter of the city, the sex of the person (H-M for man and woman in Spanish), the GSE of belonging and finally, the age of the person interviewed. The information was analyzed between July and August 2021. An inductive thematic analysis strategy of the verbatim of interviews was used, a qualitative analysis method that allows identifying thematic patterns from the data collected [38], with the support of the AtlasTi software. The thematic analysis was first organized around the categories previously identified from the SDH approach and reflected in the interview script applied to the participants (see above). Next, sub-categories and related codes were identified for each category, accompanied by verbatim participant quotes. Subsequently, emerging categories that were not contained in the previously established categories were analyzed, thus allowing the inclusion of new relevant information from the perception and experience of study subjects.

To assess the rigor of the study (credibility, dependability and confirmability), the following strategies were followed: (a) triangulation of information coming from different types of participants (from different cities, boroughs and SEG); (b) constant audit trail, by maintaining a field diary to record the ideas and experiences of the research team. This information complemented semi-structured interviews and contributed to a more coherent and trustworthy data analysis and interpretation.

### Ethical considerations

The study was designed and implemented by the universal ethical principles of scientific research: respect for people, beneficence, non-maleficence, and justice. The study contemplated an online informed consent process, ensuring that participants received relevant information about the objectives and procedures of the study and certifying their voluntary participation before the informed consent was signed. The informed consent was signed remotely by filling out a secure and encrypted online form on the Alchemer virtual platform. If the person accepted to participate, he/she could save a PDF of the informed consent that was automatically downloaded from the system. All the participants signed the informed consent. An informative PDF document was also provided to participants, with a detailed explanation of the study and their participation, as well as the contact information of the principal investigator. The data analysis process was carried out with anonymized information, preventing individual people from being recognized. Additional methodological details and general results of this study are available and freely accessible in the recently published final report [42] and policy brief [43].

## Results

51% of the study participants were women and 49% were men. 31% belonged to the most affluent socioeconomic group (SEG) ABC1 to C1B; 36% to the C2-C3 group; and 33% to the poorest D-E group (roughly a third each). 34% of the participants lived in Santiago, 33% in Valparaíso and 33% in Concepción. Table 1 describes the sociodemographic characteristics of study participants.

**Table 1:** Sociodemographic characteristics of the participants

	Santiago (n = 21)		Valparaíso (n = 20)		Concepción (n = 20)	
Age	N	%	N	%	N	%
18–24 years	6	29%	7	35%	7	35%
25–64 years	9	43%	7	35%	8	40%
65 years or more	6	29%	6	30%	5	25%
**Total**	**21**	**100%**	**20**	**100%**	**20**	**100%**
**Sex**	**N**	**%**	**N**	**%**	**N**	**%**
Women	10	48%	11	55%	10	50%
Men	11	52%	9	45%	10	50%
**Total**	**21**	**100%**	**20**	**100%**	**20**	**100%**
**SEG**	**N**	**%**	**N**	**%**	**N**	**%**
Most affluent ABC1aC1b	6	29%	6	30%	7	35%
C2-C3	9	43%	7	35%	6	30%
Least affluent D-E	6	29%	7	35%	7	35%
**Total**	**21**	**100%**	**20**	**100%**	**20**	**100%**
**Educational level**	**N**	**%**	**N**	**%**	**N**	**%**
Primary/basic	1	5%	0	0%	2	11%
Secondary education	3	14%	5	25%	5	26%
Advanced technician	5	24%	0	0%	0	0%
Incomplete university	6	29%	0	0%	0	0%
University/professional	6	29%	15	75%	12	63%
**Total**	**21**	**100%**	**20**	**100%**	**19**	**100%**
**Health insurance**	**N**	**%**	**N**	**%**	**N**	**%**
Public (Fonasa)	12	57%	5	25%	11	55%
Private (Isapre)	9	43%	13	65%	6	30%
Armed Forced and Other	0	0%	0	0%	1	5%
Doesn’t know/Doesn’t have	0	0%	2	10%	2	10%
**Total**	**21**	**100%**	**20**	**100%**	**20**	**100%**

Participants were able to describe their perceptions of main barriers and facilitators for following the COVID-19 preventive measures during the pandemic, such as social distancing, quarantines, use of masks, and hand washing; as well as control measures like COVID-19 testing and traceability. Perceptions around each of these COVID-19 preventive and control measures are presented below, according to selected dimensions of SDH as the theoretical perspective of analysis.

### Social distancing

Study participants identified several barriers to adhere to social distancing measures. They mentioned the impossibility of complying with the recommended social distance when using public transport, especially during peak hours. Similarly, they argued that it is more difficult for the poorest groups to comply with this measure than for the well-off population. They added the difficulty of social distancing when buying basic living groceries, considering that families in the lower socioeconomic groups had to leave their homes to buy in person in overcrowded settings (fairs, local supermarkets) and without the supervision of the compliance with prevention measures:

“There are people that do not have the chance to buy through delivery, it doesn´t reach their homes. So they are not being thought of, the measures are being thought from a desk, so more territorial work is needed” (E6VH,G2,24).

“In the neighborhoods where the supply is, the market…the main streets where they sell food and all that, that are like two or three, that is always full, always full. So, there is very little safeguard for that” (E17VH,G3,76).

Another barrier identified was one that the participants recognized as a typical cultural habit, in which every social gathering involved physical contact like kissing and hugging as a standard social practice:

“The culture here is of *achoclonarse* (staying close together). We greet each other with kisses, if you are talking with your friends, you are seated next to your friend, there is no distance, you are always touching each other. It’s a culture where we don’t have much distance” (E3VM,G2,35).

Young adults pointed out that, in general, they kept their distance from strangers in public spaces. However, they said they did not follow this measure when they met friends or acquaintances, since they were believed to be reliable in their self-care in relation to COVID-19:

“I believe that with people on the street, one does tend to maintain social distance, because you do not know about the other person or who they hang out with. But maybe with friends, one relaxes (…) at least in those moments I didn’t comply with, like being a meter away from my friend. I was careful with whom I got together with, like having more knowledge that the person wasn’t going out everywhere” (E10SM,G2,23).

Along with the above, the interviewees stated that the way in which the health authorities implemented social distancing did not facilitate adherence. They argued that individuals and communities should be better educated about the reasons for social distancing beyond simply informing that everyone should follow it:

“The bad communication that the authority has had about this…keep distances “just because” and if we see you, you get a fine. There is no education work, which has ultimately caused people to distance themselves from the government and its measures. What you have to generate is precisely the opposite, empathy with the population, try to raise awareness, in a good way, knowing that if you don’t comply, this will happen. Some people have the privilege of being informed, like me, but the lady who lives around the corner, that uses the mask underneath, because the elastic fell off and she hasn’t bought another…she doesn’t know where to get that information, the lady barely watches television, and on television they show every morning that there are three thousand deaths and that’s all, but there is no clear explanation of the measures” (E6VH,G2,24).

According to the people interviewed, the main facilitating aspect for adherence to this measure was the fear of contagion and death, which led some to distancing themselves from crowds and public spaces.

### Quarantines

As a facilitating aspect for adherence to staying at home, participants positively highlighted the gradual implementation of the Step-by-Step Plan. This national strategy was applied in Chile in accordance with local realities at the borough level. However, they perceived quarantines in several boroughs were excessively long and limited individuals’ compliance over time. Also, it was perceived that the quarantine experience meant having to readjust daily life, for instance having to accommodate several tasks such as working online, domestic work, childcare, and school support for children, caring for children with special needs, and caring for the elderly. Furthermore, they pointed out the significant burden that this strategy brought for women, who had to multiply their working hours to carry out all the care-related tasks. Hence, participants emphasized that one significant undesirable consequence of quarantines was that they generated an unequal negative impact of COVID-19 for women. This gender inequity against women was observed across all age groups, territories, and socioeconomic groups:

“Now, thinking about the issue of gender, regarding the topic of care, now that I think about it…as well as the role of care and worrying about one’s own and others’ health, women also carry a bigger burden, so perhaps there is also a concern on the part of women to follow the prevention measures” (E1VH,G1,28).

Another barrier to complying with quarantines identified by the participants was the economic one, linked to the need to work for subsistence even during recommended confinement. This made quarantines unrealistic, inefficient, and stressful. This meant, in many cases, a perceived loss of trust in the effectiveness of the measure at the community and population levels:

“There is no way that the people who work on the street can sustain themselves without that, without earning a living. At least not for a long time and the COVID bonus isn’t much money either. So, I am honestly stressed with the phase one (quarantine) situation, it is really hard emotionally speaking and rationally, I don’t find it so logical” (E4VM,G1,21).

Linked to quarantines, the curfew was another measure questioned by the population interviewed. People expressed that it was an excessively long and meaningless measure in terms of curbing the pandemic. One participant expressed the following:

“Yes, the curfew makes sense because it’s legal within a state of constitutional exception. But what does a curfew at nine or ten imply. The difference is that businesses close earlier, the buses leave earlier…what happens, the buses instead of getting crowded at nine, they get crowded at seven. That’s what has happened in Valparaíso, for example. People living in poverty feel that their life has been a punishment. Furthermore, you work eight hours and when you leave you want to go to the supermarket, and it’s closed. You want to go to the neighborhoods store, closed” (E6VH,G2,24).

### Use of face masks

Participants mentioned that the initial information regarding this measure provided by health authorities and healthcare teams was confusing, which made it challenging to establish face masks as a central prevention measure at the community level. Furthermore, participants mentioned the high prices of masks, which was also a barrier to its use and frequent replacement every few hours. Therefore, the use of non-disposable masks was preferred for their lower cost and durability, yet providing less or no protection against COVID-19:

“In the beginning the use of face masks was very confusing because there wasn’t an established version agreed between the government and the College of Physicians, because they said only infected people had to wear it. It was because of some kind of shortage of masks, but also the pharmaceutical companies took advantage and raised too much the price of the masks, it was too expensive” (E9SH,G2,21).

The most vulnerable groups mentioned the high price of good quality masks -which they identify as surgical masks-, explaining that they could not afford them. In addition, since they are disposable and require permanent replacement, they involve greater expense than the use of reusable masks, which are perceived as less safe:

“Because you are supposed to use the masks, the one that is useful, it’s useful twice a day, so not everybody will be able to buy that or have that sort of protection” (E2SM,G2,32).

Misusing the mask in public spaces, such as when the nose and mouth are exposed, was also recognized as a barrier. It was pointed out that the information available from health authorities regarding the use of masks needed to fully address the risks that their mishandling entails. Participants, for instance, identified street markets, public transportation, and informal settlements as places where large crowds misused masks, or simply did not use them at all:

“The other day, for example, I went to a gathering and it was like if COVID did not exist. No one, no one wore the mask. When I go to the market, I go with the mask on and everything, but I also see a lot of people, mainly the vendors, without a mask. The salespeople in the shops in my neighborhood work without a mask on” (E2SM,G2,32).

In addition, the interviewees recognized that many communities in the country did not wear the mask given the notion of collective life that prevails in their neighborhoods, where public space is conceived as an extension of private space and, therefore, not using face masks was normalized since it is not perceived as risky:

“In the camp they were giving out lunches from a common pot and the truth is that the people who came to get lunch were not wearing a mask. Sure, since they live next door, they don’t worry because they live close by” (E20VM,G3,61).

Among other elements identified as barriers to adherence to the use of masks were diminishing fear of getting COVID-19 over time, the discomfort caused by wearing a mask, and the perception of safety when socializing with family or friends. As a facilitating factor for adherence, the interviewees mentioned how quickly people included masks in their daily lives, together with the fear of contagion, which encouraged its use.

### Hand washing

Most study participants stated that since the beginning of the pandemic, they incorporated frequent handwashing into their routines, a personal hygiene practice that, before the pandemic was not part of their daily habits:

“Well, there has always been (the hand washing), but I don’t know if it’s the Chilean people, but I think it’s not an established culture, very few people washed their hands before sitting at the table before the pandemic” (E3VM,G2,35).

Among the main barriers to adherence to this hygiene routine was the gradual loss of fear of COVID-19 and the fact that people were replacing handwashing with soap and water with the use of hand sanitizer:

“We have replaced the constant hand washing with hand sanitizer. I feel that you are told to use hand sanitizer and they promote hand sanitizer, but they don’t tell you that it’s more important to wash your hands, because hand sanitizer does not replace water or soap” (E11VM,G3,20).

In terms of territorial inequalities regarding the availability of resources to prevent COVID-19, people from Valparaíso and Concepción reported that while in Santiago there was access to hand sanitizer in most public spaces, it was not as frequent in other regions:

“When I was in Santiago I realized that the situation regarding the availability of hand sanitizer is very different, since it is available in public spaces, unlike here (Concepción) where there are no dispensers or anything” (E1CH,G3,26).

Another barrier, identified mainly in Valparaíso, was the lack of access to drinking water in some more geographically remote boroughs of the city, which affected the population living in informal settlements:

“In the (Valparaíso) region there are places without water, the Petorca area…so telling them to wash your hands for thirty seconds… Sometimes in the videos they show how the water runs, it doesn’t matter, that is, that is impossible for them” (E3VM,G2,35).

Fear of contagion was identified as the main facilitator to compliance with handwashing recommendations. They mentioned the inclusion of this measure into their everyday routines and a greater awareness of the importance of handwashing to avoid infections, especially among older adults, since they remembered previous health crises in the country, such as cholera:

“I believe that the facilitator, precisely, I think it is fear and not wanting to get infected, people are forced to wash their hands, use hand sanitizer, carry their own bottle, arrive home and wash their hands” (E6VH,G2,24).

### COVID-19 testing (PCR)

In relation to the PCR testing for COVID-19, most interviewees said they were in favor of taking the test, and several confirmed having taken it both for presenting symptoms and for having been in close contact with a confirmed case COVID-19. On the one hand, the participants reported that access to PCR testing was faster and more accessible through the private health system; however, they identified the high cost of the test in the private system as a barrier for those who did not have a work contract considering that at the beginning of the pandemic, some workplaces paid for the tests. On the other hand, they reported that in the public system, access to PCR was slow and testing centres were often overcrowded, making them a potential source of infection according to the interviewees:

“When I arrived there, people were very sick, they couldn’t breathe, and people like that were just going to do the PCR test. So when I arrived I walked into a room in the primary health care facility here in Lo Prado, and it was a closed room, there was only one window open, and they put me there and suddenly four people entered, and those people really were in bad shape” (E18SH,G3,19).

Another barrier of the public health sector about taking PCR tests identified by the participants was the requirement to present symptoms to be eligible for one, which left those suspected of being asymptomatic without testing. One positive aspect of the public health sector that participants highlighted was the reach-out activities carried out around the country providing free tests. Nevertheless, they warned about the need to improve access to the information regarding the places, days and times where they were carried out, since the main information channels identified by the participants were social networks, excluding those who do not use them:

“I think that with social media, on the internet it’s easy to find information. I think it is difficult, mainly for people who do not have access to the internet, especially those people who do not have internet literacy” (E10SM,G2,23).

Participants also identified fear as a barrier to taking the test due to the risk of being discriminated against for having COVID-19 and not being able to work for daily wages. In this context, they observed that, given the need to work to make money to live, people with a positive PCR left their homes anyway:

“It happened a lot that people didn’t go to get the test, or they took it when they were very sick, or if it came out positive, they hid it from all the other people with whom they lived because they were the only source of income, or the others had to work and things like that” (E3SH,G2,25).

Many participants said they had taken the test, some more than once, both for having symptoms and for being in close contact with someone infected. In addition, experiences related to COVID-19 testing were positive.

### Traceability

Although most participants considered traceability crucial for controlling COVID-19, they reckoned that it was difficult to implement given the need for coordination between the different state institutions involved. In turn, they detected a lack of rigor from the general population in communicating when they had been in close contact with a COVID-19 positive case. In this sense, some participants even mentioned that they were not sure if, when the time came, they would give the name of all the people with whom they had been in contact with, given the restriction of freedoms that this implied in providing this information to health authorities:

“It’s complicated to be a close contact (to someone with COVID-19) today, it takes away your freedom, they force you to take a PCR test, they keep you “monitored”, it isn’t a “big brother” either, but they keep you there. That is why many people wait until they feel really bad to have the test, because in the end, it takes away your freedom. So in general, people don’t talk too much or they don’t give out 100% of their close contacts” (E9SH,G2,21).

As a facilitating factor for traceability, despite reservations towards freedom limitations, the interviewees expressed that this measure is essential to curb the pandemic, leading to adherence among some.

In all, the interviewee´s experiences regarding the different health measures discussed in this study allow us to understand how the social determinants of health are involved, especially concerning barriers to adherence. Belonging to a lower socioeconomic group, facing job insecurity and needing to leave home to generate a daily income, being a woman, not having access to basic supplies such as drinking water, and overcrowding were the main factors affecting the participants’ compliance with different preventive and control measures against COVID-19. In addition to this, structural socio-cultural elements like community practices of sharing personal items, kissing and hugging, as well as social interactions in public spaces in low-income boroughs (informal settlements, overcrowded street markets, lack of readily accessible hand sanitizer in public spaces, and others) proved their relevance to how individuals understood and related to COVID-19 preventive and control measures. Furthermore, gender inequity surfaced due to some of these measures, as women faced the challenges of multitasking and work overload over time. Regarding the healthcare system, although there were efforts to increase the number of hospital beds through the Integrated Health Network, some other aspects were sidelined. As participants emphasize, the access to COVID-19 testing was unequal between the private and public healthcare systems. While in the private system access was fast, and results were ready in a short period of time, in the public system the process was more bureaucratic and slower, with multiple requirements to be eligible for a test such as having symptoms. This also made public health care facilities overcrowded, which people perceived as a barrier due to the fear of getting infected by being close to crowds.

The main results of the study are summarized in Table 2.

**Table 2:** Barriers and facilitators for adherence to COVID-19 prevention measures from a SDH perspective

Measure	Barriers	Facilitators	SDH involved
**Social distancing**	Having to use public transportation for commutes Not being able to order groceries online because of geographical location, leading to going to overcrowded markets Standard social practices such as kissing and hugging Trusting friends’ and acquaintances’ compliance of measures outside of the friend group Lack of reliable and relatable information	Being able to order groceries online Fear of illness and death	Socioeconomic level Geographical location Cultural practices Age Government policies
**Quarantines**	Length of mandatory quarantines and curfews Gender imbalances and expectations that women would have to work and perform care at the same time Informal labor and loss of daily wages	Gradual implementation of the Step-by-Step Plan	Lifestyle Government policies Gender Socioeconomic level Employment
**Face masks**	Lack of reliable and relatable information Price Misuse Beliefs and practices around socializing Diminishing fear of getting infected Discomfort	Inclusion as part of daily life Fear of illness and death	Lifestyle and beliefs Social practices Government policies Socioeconomic level
Hand washing	Diminishing fear of getting infected Replacing hand washing with using hand sanitizer Geographical disparities regarding availability of hand sanitizer in public places Lack of access to clean water	Inclusion as part of daily life Fear of illness and death Previous experience with epidemics	Lifestyle and beliefs Age Geographical location Access to clean water
COVID-19 PCR testing	Eligibility requirements of the public healthcare system Overcrowded public testing centres Cost of private testing Informal labor and loss of daily wages Lack of easily accessible information regarding reach-out activities with free testing Risk of discrimination	Positive attitudes and experiences towards testing Access to private testing Having a formal work contract Reach-out activities with free testing	Attitudes and beliefs Government policies Socioeconomic level Healthcare system Literacy Employment
Traceability	Lack of intersectoral coordination Lack of reliable reporting Restriction of freedom and lack of privacy	Positive attitude towards the usefulness of traceability	Attitudes and beliefs Government policies

## Discussion and conclusions

The present study explored perceived barriers and facilitators for adherence to the COVID-19 prevention and control measures promoted by the health authorities in Chile, among the population aged 18 and over, in three large cities in 2021, from the SDH perspective. According to the study findings, people men and women from different age groups, socioeconomic levels and with different health insurance reported multiple barriers to adhering to preventive COVID-19 measures. Likewise, in accordance with the literature, the findings show that the wealthiest socioeconomic groups are more likely to adhere to these measures, unlike the most vulnerable groups -especially those who do not have a formal job and must go out to the street for daily wages [11, 13, 19, 30]. Additionally, it is clear from the results that several SDH are at play in determining non-adherence to the measures, such as living in overcrowded conditions, precarious working conditions, loss of employment, the use of public transport as the only commuting alternative, limited access to basic supplies such as drinking water, among others [27, 28]. In addition to this, cultural aspects that affect non-adherence to prevention measures are identified, for example, community life taking place in public spaces, where practices usually reserved for private spaces in the context of the pandemic, such as not using face masks or forgoing social distancing, are reproduced.

The perception that the State has favored measures that are perceived as punitive, including prolonged quarantines and curfews, also affect adherence to protection measures. For this reason, the study participants expressed the need for a change in the authorities’ strategies, in order to face the deep fatigue and emotional exhaustion generated by the pandemic. Furthermore, prevention and control strategies should consider the diverse realities faced by the Chilean population as well as the SDH that specifically affects specific groups, considering that not everyone faces equal conditions to deal with a health crisis of this magnitude. These gaps lead to inequities in the response to the pandemic, with vulnerable groups being the most exposed to contagion and death from COVID-19. Consistent with the above, the SDH approach that considers some of the dimensions identified in this study as minimum dimensions of approach, such as age, sex, living in an urban or rural area, socioeconomic level, and type of health insurance, is central to efforts to promote greater adherence to prevention and testing measures. According to the existing evidence at international level, other dimensions of the SDH that could be considered are ethnicity and migration [3], which were not included in this study. Likewise, it is relevant to introduce a gender approach to containment strategies given the inequities produced and exacerbated during the pandemic, where it is identified that economic and care responsibilities within households in times of crisis fall mainly on women.

It is important to highlight that only some facilitators for adherence to prevention measures by the study participants were identified. Fear of contagion and death is recognized as the primary motivation for compliance, which reveals the distance between the standards proposed by the health authorities and the self-care practices of the population as responses to health crises such as the COVID-19 pandemic.

As a general recommendation, we suggest that strategies focus on facilitating the integration of prevention measures in the daily habits of individuals and communities through participatory actions involving populations and authorities from a rights-based, intercultural and gender approach that takes into consideration and respects the cultural and social diversity of the country. This is highly significant because of the uncertainty surrounding the medium-term evolution of the COVID-19 pandemic and the challenge that new health crises may raise at the local and global levels.

This study presents both strengths and limitations. First, regarding strengths, it is the first study of this kind in Chile and using the qualitative paradigm allows for an in-depth exploration of the phenomenon from the voice of critical actors. Additionally, using the SDH approach adds analytical depth and unveils the structural dimensions of the inequities perceived. Second, with regards to limitations, carrying out the interviews during mandatory quarantines made it impossible to access the territories and the people interviewed in person, limiting a direct appreciation of the impact of the preventive measures of the pandemic in the contexts studied. Another limitation is that the sample was preferably made up of residents of urban areas, leaving rural sectors less represented in the study.

The results of this study have direct implications for public health as they are relevant for the construction and adjustments of health policies and actions in the face of crises such as the COVID-19 pandemic. Furthermore, promoting future qualitative research on the topic would allow the collection and systematization of further valuable information about strategies to face the pandemic to bridge adherence gaps and ensure the acceptability of the measures taken for the diverse groups in our society.

## Data Availability

The data that support the findings of this study are available on request from the corresponding author A.O. The data are not publicly available due to them containing information that could compromise research participant privacy/consent.
